# Modeling of Droplet Generation in a Microfluidic Flow-Focusing Junction for Droplet Size Control

**DOI:** 10.3390/mi12060590

**Published:** 2021-05-21

**Authors:** Ali M. Ibrahim, Jose I. Padovani, Roger T. Howe, Yasser H. Anis

**Affiliations:** 1 Mechanical Design and Production Department, Faculty of Engineering, Cairo University, Giza 12613, Egypt; ali.ibrahim2@mail.mcgill.ca; 2 Department of Electrical Engineering, Stanford University, Stanford, CA 94305, USA; jose.padovani@medtronic.com (J.I.P.); rthowe@stanford.edu (R.T.H.)

**Keywords:** microfluidics, droplet generation, droplet modulation, emulsions

## Abstract

In this paper, we study the parameters that affect the generation of droplets in a microfluidic flow-focusing junction. Droplets are evaluated based on the size and frequency of generation. Droplet size control is essential for microfluidic lab-on-a-chip applications in biology, chemistry, and medicine. We developed a three-dimensional numerical model that can emulate the performance of the physical system. A numerical model can help design droplet-generation chips with new junction geometries, different dispersed and continuous phase types, and different flow rates. Our model uses a conservative level-set method (LSM) to track the interface between two immiscible fluids using a fixed mesh. Water was used for the dispersed phase and mineral oil for the continuous phase. The effects of the continuous-to-dispersed flow rate ratio ($Q_{o}/Q_{w}$) and the surfactant concentration on the droplet generation were studied both using the numerical model and experimentally. The numerical model was found to render results that are in good agreement with the experimental ones, which validates the LSM model. The validated numerical model was used to study the time effect of changing $Q_{o}/Q_{w}$ on the generated droplet size. Properly timing when the flow rates are changed enables control over the size of the next generated droplet, which is useful for single-droplet size modulation applications.

## 1. Introduction

Over the last two decades, droplet-based microfluidics has become a rapidly growing area of research due to its wide range of applications. It has proved to offer significant advantages to different fields, including biology, chemistry, and medicine [1,2]. One major advantage is that microdroplets use very small amounts of samples and reagents, which enable different chemical and biological processes to be inexpensive, more efficient, faster, and automated [3]. Examples of applications include the use of microdroplets as chemical microreactors that carry out reactions at a very small scale [4], or in performing high-throughput biological screens and sensitive assays [5], rapid DNA analysis [6], and protein crystallization [7]. Determining the parameters affecting droplet formation is therefore important, since many of these applications require precise control of droplet size.

The droplets are generated from the intersection of two immiscible fluids (a dispersed and a continuous phase). Different microfluidic systems have been developed to generate monodispersed droplets using T-junctions, Y-junctions, and flow-focusing cross-junctions [8]. Those with flow-focusing junctions are the most common due to their ability to generate smaller droplets with better size control and uniformity [9,10]. In a flow-focusing junction, the intersection of two oil channels and one aqueous channel is used to generate monodispersed aqueous droplets. The process of droplet formation in microfluidic junctions depends on numerous parameters including the junction geometry, the wetting properties of the microchannel, and the flow rates and viscosities of the dispersed and continuous phases, in addition to the interfacial tension between the two fluids [11].

Droplet size modulation (DSM) can be performed in different ways, including using syringe pumps to control the flow-rate ratio between the oil and aqueous channels ($Q_{o}/Q_{w}$), where higher ratios lead to smaller droplets [12]. Faster, active DSM methods have been proposed including electrical [13], acoustic [14], mechanical [15], thermal [16], magnetic [17], and using electropermanent magnets [18]. It is thus important to numerically model the droplet generation process such that it predicts the effects of the different parameters affecting the generation. The availability of a numerical model can also help in the process of designing droplet-generation chips with new junction geometries, different dispersed and continuous phase types, and different flow rates.

Many numerical modeling techniques have been reported, including lattice-Boltzmann methods (LBMs) and level-set methods (LSMs). Three-dimensional models based on LBMs have been used to model droplet formation in flow-focusing junctions [19,20,21]. The LBM model results reveal that both the size of the generated droplets and the flow regimes in microfluidic flow-focusing cross-junctions are affected by the junction geometry, the flow rates of dispersed and continuous phases, and the capillary number [19]. Limitations in the LBM model can include (1) the existence of unphysical spurious currents at the liquid–liquid interface, which lead to numerical instability; (2) the difficulty to separately adjust important parameters such as the interfacial tension, due to the coupling between LBM properties; and (3) the limited density and viscosity ratios achievable [22]. Alternatively, level-set methods (LSMs) have been used to model droplet generation [23,24,25,26,27]. The LSM is a simple method used to track moving interfaces using a fixed mesh in plane or space [28]. Using an LSM with a T-junction, Bashir et al. reported that the wetting properties of the junction walls affect the frequency of droplet generation as well as the droplet size; furthermore, increasing the interfacial tension between the two fluids increases the droplet sizes [23]. An LSM was used to study the effect of geometric and fluidic parameters on the droplet length and frequency in flow-focusing junctions [25,26]. However, these studies were not validated. Mottaghi et al. mixed artificial neural networks and fuzzy inference systems, to study the parameters that affect the droplet size, numerically and experimentally [29].

In this paper, we use a conservative level-set method (LSM) to create a three-dimensional numerical model for the generation of droplets in a microfluidic device with a flow-focusing junction. Oil is used as the continuous phase with water as the dispersed phase. We use the model to study the effects of the flow rate ratio and the interfacial surface tension on both the steady-state and transient generated droplet parameters, and compare our findings to those achieved experimentally. The parameters of most interest include the droplet diameter and the frequency of droplet generation. The frequency of generation is represented by the spacing between two consecutive droplets. The model is used to study the transient effect of the change in the continuous phase flow rate on the generated droplet size.

## 2. System Description

**Figure 1:**
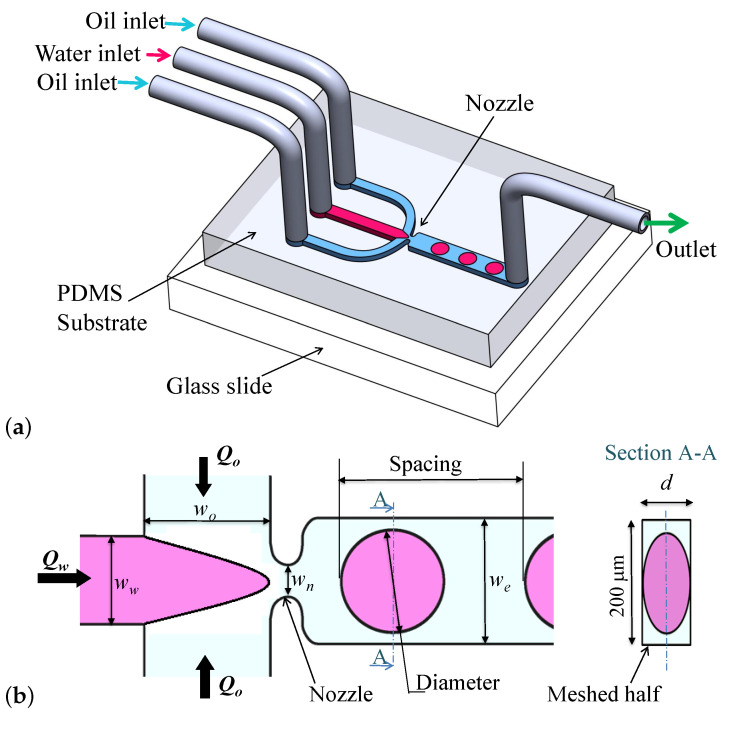
Schematics for generation of water droplets in oil using a flow-focusing cross-junction. (**a**) 3D schematic for the device, (**b**) 2D schematics showing the microchannel dimensions.

The microfluidic droplet generation device includes a polydimethylsiloxane (PDMS) substrate, bonded to a glass slide, with three inlet ports and one outlet, as shown in Figure 1. The microfluidic circuit has one central inlet channel of width $w_{w}$, two lateral inlets each of width $w_{o}$, and one outlet of width $w_{e}$. The device channels have a uniform depth *d* across the entire circuit. The dispersed water phase is injected through the main inlet at a flow rate $Q_{w}$, while the continuous oil phase is injected through each lateral inlet at a flow rate $Q_{o}$.

### 2.1. Droplet Generation

**Figure 2:**
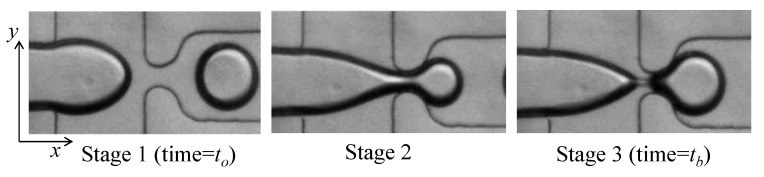
Micrographs showing the stages of droplet generation.

The droplet generation process can be described by three repeated stages, shown in Figure 2. First, the flow of the dispersed phase expands in the *x* and *y* directions until the nozzle is reached. Second, the dispersed phase passes through the junction nozzle into the main channel. Third, the tip of the dispersed phase expands into the main channel, and a neck is created in the nozzle that then shrinks until it collapses. The droplet is released afterwards and the dispersed phase retracts away from the nozzle (See Figure 2: Stage 1). Typically, the three-dimensional shape of the generated droplets is not spherical, except for when the droplet diameters are smaller than the channel depth *d*. Droplets have a spheroid shape with circular cross-sections in the planes parallel to the glass slide, as shown in Figure 1b.

### 2.2. Experimental Setup

Oil and water were injected into the microfluidic device inlet ports using 22-gauge steel pins and Tygon tubing (Instech Laboratories, Plymouth Meeting, PA, USA), connected to two dual-syringe pumps (Pump 11 Pico Plus Elite, Harvard Apparatus, MA, USA). Mineral oil (MilliporeSigma, MO) was used as the continuous phase. Span80 surfactant (Millipore Sigma, St. Louis, MO, USA) was added to the oil, at concentrations $S_{c}$ varied between 0.5% and 1% (*wt*/*wt*) to prevent droplet coalescing and reduce the interfacial tension between oil and water [18]. The generated droplets were imaged using a camera–microscope system that included a high-speed sCMOS camera (Andor${}^{TM}$ Zyla 4.2, South Windsor, CT, USA), connected to an inverted microscope (Eclipse Ti-E, Nikon, Tokyo, Japan) with a 4× objective (CFI Plan Achromat UW 4×, Nikon). A computer program, custom-written in LabView (National Instruments, Austin, TX, USA), was used to measure the mean values and standard deviations for both the droplet diameter and the spacing between two consecutive droplets.

### 2.3. Device Fabrication

The microfluidic devices were fabricated in polydimethylsiloxane (PDMS) (Sylgard 184, Dow Corning, MI, USA) using standard lithography techniques [30]. The mold was generated by patterning microchannels on a clean silicon wafer using SU-8 negative photoresist (MicroChem, Newton, MA, USA). The photoresist was spun at 2000 RPM for 45 s, exposed to UV light through a 40,000 dpi transparency mask (IGI, Minneapolis, MN, USA), and developed according to the manufacturer’s protocol. The silicon wafers were silanized with chlorotrimethylsilane (Sigma-Aldrich, St. Louis, MO, USA) for 45 min inside a desiccator. PDMS was cast against the mold using a 10:1 ratio with its crosslinker after degassing to remove air bubbles. The PDMS was cured at 60 °C for 2 h before use. After curing, the chips were removed from the PDMS, the inlet and outlet ports holes were punched, and the chip was bonded to a clean cover glass using air-plasma activation of both the PDMS and cover glass in a plasma cleaner (Harrick Plasma PDS-32G, Ithaca, NY, USA).

## 3. Modeling

A three-dimensional numerical model is presented using two-phase level-set methods (LSMs) to simulate the generation of water droplets in a continuous oil phase. The model, once validated experimentally, can be used to study the effect of the different parameters affecting the droplet generation process. The outcome of these studies can help in the design of future drop-generation devices.

### 3.1. Governing Equations

Here, we use the modified conservative LSM, developed by Olsson and Kreiss [31], in which the interface between the aqueous sample and the oil is represented by the 0.5 level-set of a function ϕ. The function ϕ changes smoothly over a constant thickness transition layer from zero (at the continuous phase) to one (at the dispersed phase). Motion of the continuous–dispersed phase interface is governed by Equation (1), coupled with the incompressible Navier–Stokes and continuity Equations (2) and (3):

(1) $$\frac{\partial \phi }{\partial t}+u\cdot \nabla \phi =\gamma \nabla \cdot \left[\varepsilon \nabla \phi -\phi \left(1-\phi \right)\frac{\nabla \phi }{\left|\nabla \phi \right|}\right],$$

(2) $$\rho \frac{\partial u}{\partial t}+\rho \left(u\cdot \nabla \right)u=\nabla \cdot \left[-pI+\mu (\nabla u+{\left(\nabla u\right)}^{T})\right]+F_{st},$$

(3) ∇·u=0,

where u is the velocity (m/s), ρ is the density (kg/m${}^{3}$), *p* is the pressure (N/m${}^{2}$), μ is the dynamic viscosity (Ns/m${}^{2}$), $F_{st}$ denotes the surface tension force acting at the interface between the two phases (N/m${}^{3}$), and γ (m/s) is a parameter used for reinitialization that is usually set to the maximum magnitude of the velocity in the model. The parameter ε (m) is used to control the interface thickness over which ϕ changes from 0 to 1. A suitable value for ε is half the characteristic mesh size $h_{c}$ ($\varepsilon =0.5h_{c}$). The density ρ and dynamic viscosity μ are calculated from the equations:

(4) $$\begin{array}{c} \rho =\rho_{o}+\left(\rho_{w}-\rho_{o}\right)\phi \end{array}$$

(5) $$\begin{array}{c} \mu =\mu_{o}+\left(\mu_{w}-\mu_{o}\right)\phi \end{array}$$

where $\rho_{o}$ and $\rho_{w}$, $\mu_{o}$ and $\mu_{w}$ are the densities and viscosities of the continuous and dispersed phases, respectively.

### 3.2. Geometry and Model Parameters

The microfluidic device used in this work was designed to have a cross-junction with the dimensions shown in Figure 1b, where $w_{o}=w_{e}=$ 200 μm, $w_{w}=$ 155 μm, $w_{n}=$ 50 μm, and d= 55 μm. In the numerical model, we took advantage of the symmetry to reduce the number of mesh elements. Thus, only the upper half of the device and the droplets were considered, which is represented by the half-device on the left of the dash-dotted line, shown in Section A-A of Figure 1b. This reduced both the number of mesh elements and the simulation time by one-half. For our 55 μm deep channels, the model only used 27.5 μm in thickness in order to generate only half-droplets. An entrance length of 4200 μm was selected for the inlet of the dispersed phase, while entrance lengths of 1250 μm were selected for each of the two lateral inlets. The gauge pressure at the outlet was assumed zero. For the dispersed phase we used water with $\rho_{w}$ = 1000 kg/m${}^{3}$ and $\mu_{w}$ = 0.00105 Pa.s. For the continuous phase, we used mineral oil with $\rho_{o}$ = 800 kg/m${}^{3}$ and $\mu_{o}$ = 0.153 Pa.s. Constant velocity profiles were applied on the respective inlets of water and oil. The surface tension coefficient was adjusted to σ=3 mN/m, which corresponds to a Span80 surfactant concentration of 0.5%. Wetted walls were used for the microchannel with a contact angle of 135° and a slip length β=0.1$h_{c}$. The friction force $F_{fr}$ between the walls and the fluid–fluid interface is represented by:

(6) $$F_{fr}=\frac{\mu }{\beta }u$$

**Figure 3:**
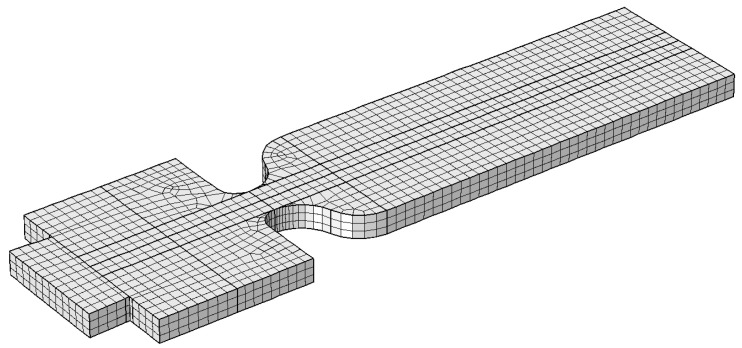
The three-dimensional mesh used for the numerical model. Number of mesh elements is 5208.

We used 5208 hexahedral elements to create the mesh with a maximum element size $h_{c}=11$ μm, shown in Figure 3. The numerical model was solved using a computational fluid dynamics (CFD) package (COMSOL Multiphysics^®^, Stockholm, Sweden).

### 3.3. Grid Dependence

**Figure 4:**
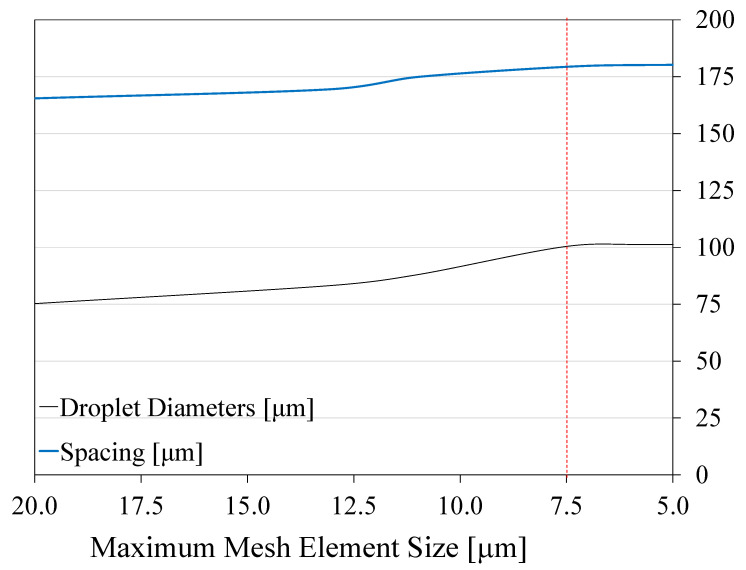
Results from grid dependence study showing the effect of the mesh element size $h_{c}$ on the droplet diameter and droplet spacing.

A grid dependence study was performed to assess the effect of the mesh element size $h_{c}$ on the simulated droplet diameter and spacing. The study was performed at constant flow rates of $Q_{w}=0.5$ μL/min and $Q_{o}=1.2$ μL/min. The interfacial tension coefficient σ was selected as 3 mN/m. The maximum element size $h_{c}$ in the numerical model was decreased from 20 to 5 μm. The dependence of the generated droplet diameter and spacing on $h_{c}$ is presented in Figure 4, which reveals that the effect of decreasing the mesh element size on the droplet diameter and spacing diminishes for $h_{c}<$ 7.5 μm. Because simulations using $h_{c}<$ 7.5 μm were found to be computationally expensive, an element size $h_{c}=11$ μm was selected as it was found to produce reasonably accurate results, but with less computational cost.

## 4. Model Validation

The effects of the flow rate ratio ($Q_{o}/Q_{w}$) and the surfactant concentration ($S_{c}$) on the droplet generation were studied both experimentally and numerically using the conservative two-phase level-set method (LSM) model, described earlier. The investigated droplet parameters included the droplet diameter and spacing, which represent the droplet size and frequency of generation, respectively.

### 4.1. Flow Rate Effect on Droplet Generation

Two cases were considered: (1) water flow rate kept constant at $Q_{w}=0.5$ μL/min, while varying the oil flow rate ($Q_{o}$) between 0.5 and 2 μL/min; and (2) $Q_{w}$ kept constant at 0.2 μL/min, while varying $Q_{o}$ between 0.3 and 1.2 μL/min. The surfactant concentration $S_{c}$ was kept constant at 0.5% (*wt*/*wt*). Experiments were performed at a number of discrete $Q_{o}$ values that cover each range.

**Figure 5:**
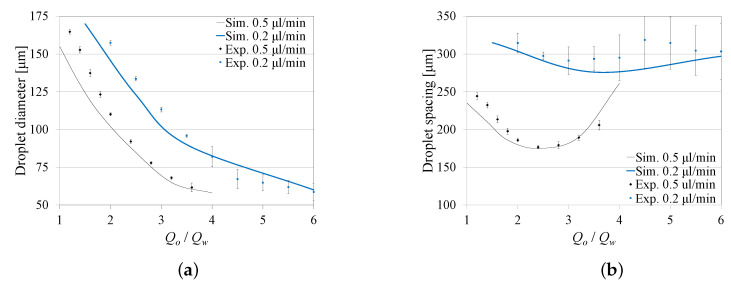
Effect of the flow rate ratio $Q_{o}/Q_{w}$ on the droplet diameter and spacing for different dispersed phase flow rates ($Q_{w}$). (**a**) Effect of $Q_{o}/Q_{w}$ on diameter. (**b**) Effect of $Q_{o}/Q_{w}$ on spacing.

Figure 5a,b show the effect of $Q_{o}/Q_{w}$ on the droplet diameter and the spacing between two consecutive droplets, obtained both numerically and experimentally. The experimental results are represented by mean values and standard deviations. The figures show a satisfactory agreement between the numerical results and those obtained experimentally, which validates our numerical model. The difference between both results could possibly be caused by a number of factors that were ignored in the system model, including the compliance in the PDMS microfluidic device, fluid leakage, temperature variations, and fabrication errors. The results in Figure 5a,b were also found to be in agreement with those reported in [23,24,25,29].

Figure 5a,b also show that at a constant $Q_{o}/Q_{w}$, both the droplet diameter and spacing decrease with the increase in $Q_{w}$. At a constant $Q_{w}$, the droplet diameter decreases with the increase in both $Q_{o}$ and $Q_{o/w}$. However, an increase in $Q_{o}$ and $Q_{o}/Q_{w}$ causes the droplet spacing to decrease until a minimum spacing value is reached, corresponding to the maximum frequency of generation, and afterward it starts increasing again. This minimum spacing value is caused due to the increase in the rate of decrease in the droplet size with $Q_{o}/Q_{w}$, which is found to be maximum near $Q_{o}/Q_{w}$ = 2.5 for $Q_{w}=0.5$ μL/min.

### 4.2. Surfactant Concentration Effect on Droplet Generation

Two cases were considered: (1) at surfactant concentration $S_{c}=0.5\%$ (*wt*/*wt*), which corresponds to σ=3 mN/m, while varying $Q_{o}$ between 0.9 and 2 μL/min, and (2) At $S_{c}=1\%$ (*wt*/*wt*), which corresponded to σ=0.4 mN/m, while varying $Q_{o}$ between 0.5 and 1.4 μL/min. Experiments were performed at a number of discrete $Q_{o}$ values that cover each range, while keeping $Q_{w}$ constant at 0.5 μL/min. The experimental results are represented by mean values and standard deviations.

**Figure 6:**
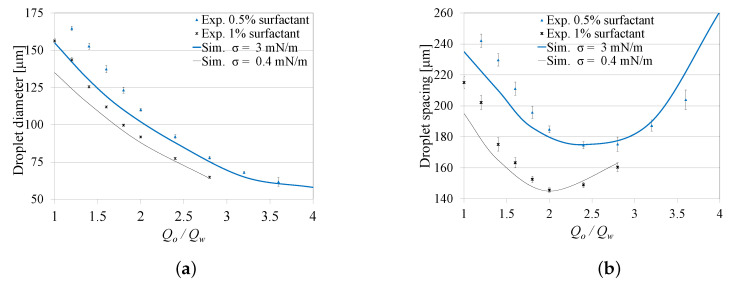
Effect of the flow rate ratio $Q_{o}/Q_{w}$ on the droplet diameter and spacing for different surfactant concentrations. (**a**) Effect of $S_{c}$ on diameter. (**b**) Effect of $S_{c}$ on spacing.

Similar to Section 4.1, Figure 6a,b show a satisfactory agreement between the numerical results and those obtained experimentally, representing the effect of $Q_{o}/Q_{w}$ on the droplet diameter and the spacing. The figures also show that at a constant $Q_{o}/Q_{w}$, both the droplet diameter and spacing increase with the decrease in $S_{c}$, corresponding to a decrease in the interfacial tension (σ). The effect of the interfacial tension on the frequency of droplet generation was found to be in agreement with those reported in [25,26].

### 4.3. Transient Effect of Flow Rate Change on Droplet Size

In droplet size modulation (DSM) applications, the $Q_{o}/Q_{w}$ ratio is typically changed so as to affect the size of the next generated droplets. When the $Q_{o}/Q_{w}$ is changed (at an initiation time $t_{c}$), the size of the next generated droplet depends upon its generation stage. The generation stages are described in Section 2.1 and are defined by when $t_{c}$ occurs relative to $t_{0}$, the time at which the tip of the dispersed phase is about to enter the nozzle, and $t_{b}$, the time when the droplet breaks up (see Figure 2). This makes the prediction of the next generated droplet size challenging, which affects single-droplet size modulation applications.

We used the numerical model to investigate the effect of the change in $Q_{o}/Q_{w}$ on the size of the next generated droplet. This change is defined by a time function that takes place at the time $t_{c}$. A simple ramp time function is used, where $Q_{o}$ is changed between two values, $Q_{o-min}$ and $Q_{o-max}$, while keeping $Q_{w}$ constant. The duration of the $Q_{o}/Q_{w}$ change (Δ) depends upon the difference between $Q_{o-min}$ and $Q_{o-max}$, in addition to the type of pump used. When changing the flow rates using syringe pumps, the duration Δ can last several seconds, while using electropermanent magnets for faster flow control can reduce Δ down to 100 μs [18].

**Figure 7:**
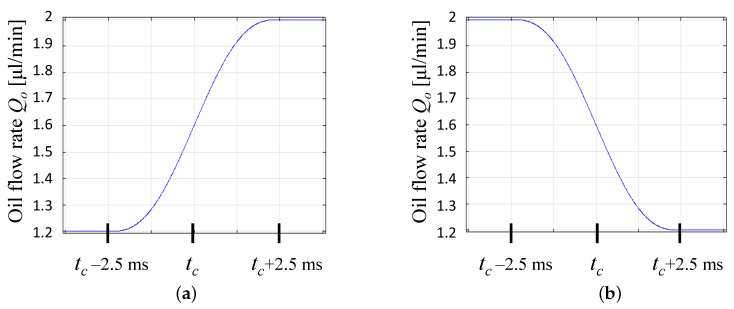
Time functions for (**a**) the increase of of $Q_{o}$, (**b**) the decrease of $Q_{o}$.

As a proof of concept, we used the ramp time functions shown in Figure 7, where $Q_{o-min}=$ 1.2 μL/min and $Q_{o-max}=$ 2 μL/min, while keeping $Q_{w}$ constant at 0.5 μL/min. A surfactant concentration $S_{c}=0.5\%$ (*wt*/*wt*) was selected, which corresponds to σ=3 mN/m. Here, the duration between the beginning of the droplet buildup until breakup was approximately 25 ms. Therefore, the duration of the $Q_{o}/Q_{w}$ change was arbitrarily selected as Δ= 5 ms, as shown in Figure 7.

**Figure 8:**
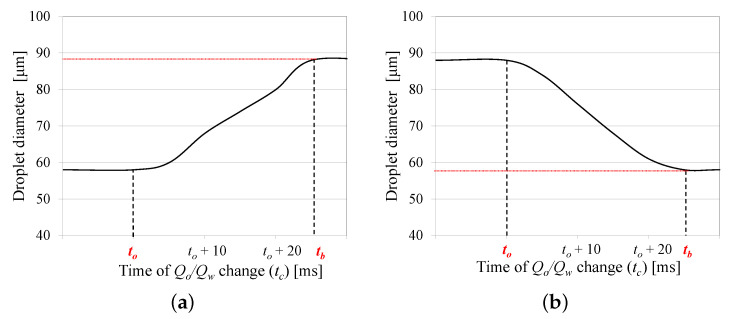
The effect of the time of the increase or decrease in oil flow rate ($t_{c}$) on the size of the first generated droplet for (**a**) the increase of of $Q_{o}$ (**b**) the decrease of $Q_{o}$. The red dotted line represents the droplet size if $Q_{o}/Q_{w}$ does not change.

Figure 8a shows the effect of the initiation time $t_{c}$ on the generated droplet diameter when increasing $Q_{o}$ from $Q_{o-min}$ to $Q_{o-max}$ (see Figure 7a). This figure is divided into three parts: (1) when $t_{c}<t_{0}$, the diameter of the next generated droplet drops to 58 μm (equivalent to a steady oil flow rate $Q_{o-max}=$ 2 μL/min in Figure 6a); (2) when the increase starts at the time when the droplet is about to break up ($t_{c}>t_{b}$), the released droplet diameter will not be affected by this flow rate change, resulting in a droplet with an 88 μm diameter (equivalent to a steady oil flow rate $Q_{o-min}=$ 1.2 μL/min); (3) when $t_{c}$ falls between $t_{0}$ and $t_{b}$, here the droplet diameter will have a value between 58 μm and 88 μm. When the system is subjected to a flow rate decrease, as represented by the time function in Figure 7b, a similar behavior occurs. Figure 8b shows the effect of $t_{c}$ when decreasing $Q_{o}$ from $Q_{o-max}$ to $Q_{o-min}$.

Therefore, to maximize the size of the next generated droplet, it is preferable to perform the flow rate change $t_{c}<t_{0}$. Changing the oil flow rate at $t_{c}>t_{0}$ will only partially impact the size of the next generated droplet. It is worth noting that the duration of flow rate change (Δ) needs to be much smaller than ($t_{b}-t_{o}$), which is affected by the droplet spacing.

## 5. Conclusions

We used a conservative two-phase level-set method to create a three-dimensional numerical model that simulates the generation of water-in-oil droplets in a microfluidic flow-focusing cross-junction. The model was experimentally validated and used to study the effects of a number of parameters that affect the generated droplet diameters and the spacing between consecutive droplets.

The study reveals that an increase in the oil-to-water flow rate ratio leads to a decrease in the droplet diameters. Furthermore, it also leads to a decrease in the droplet spacing until a specific lowest value is reached, after which the spacing increases again. Results also indicate that decreasing the water flow rate, while keeping the oil flow rate constant, increases both the droplet diameter and spacing. At a constant flow rate ratio, both the droplet diameter and spacing increase with the decrease in the surfactant concentration, which corresponds to a decrease in the interfacial tension.

When the flow rate ratio is changed, the resulting droplet diameters depend upon a number of factors, including (1) the rate of change, (2) the time at which the change starts, and (3) the position of the dispersed phase relative to the nozzle when the change starts. The model of how flow parameters affect the droplet size and spacing is very important for the design of systems to modulate droplets in flow-focusing junctions. In DSM applications, it is preferable to change the flow rate ratio right before the time when the tip of the dispersed phase is about to enter the nozzle.

## Abbreviations

DSM	Droplet size modulation
LBMs	Lattice–Boltzmann methods
LSMs	Level-set methods
PDMS	Polydimethylsiloxane
